# Research on construction workers’ job well-being and unsafe state: From the perspective of Maslow hierarchy of needs

**DOI:** 10.1097/MD.0000000000043605

**Published:** 2025-08-01

**Authors:** Qi Liu, Zhengqing Zhong, Yaxin Li

**Affiliations:** a School of Marxism, Wuhan University of Science and Technology, Wuhan, Hubei, China; b Institute of Safety and Emergency, Wuhan University of Science and Technology, Wuhan, Hubei, China.

**Keywords:** DEMATEL-ISM model, job well-being, Maslow hierarchy of needs, unsafe state

## Abstract

To enhance construction workers’ job well-being and reduce unsafe behaviors, this paper introduces Maslow hierarchy of needs theory. It deeply analyzes the intrinsic relationship between construction workers’ job well-being and their unsafe conditions (both psychological and physiological states) and establishes a framework for understanding how job well-being impacts unsafe conditions. An indicator system for factors affecting construction workers’ job well-being is constructed based on 5 dimensions: living conditions, working conditions, rights protection, safety development (stress), and career advancement. The DEMATEL-ISM model is utilized to identify key influencing factors and explore the hierarchical structural relationships among these factors. The results indicate a direct correlation between the level of job well-being among construction workers and their unsafe conditions. Key factors impacting job well-being include income, safety assurance, working environment, working hours, and work-related stress, which warrant focused attention.

## 1. Introduction

Data shows that approximately 2.78 million people die each year due to work-related factors. The economic costs associated with workplace injuries, illnesses, and accidents are enormous, consuming about 4% of global GDP.^[1]^ Against this grim backdrop, the National Institute for Occupational Safety and Health has launched the Total Worker Health program, which integrates the protection against work-related safety and health hazards with injury and disease prevention initiatives, aimed at improving overall worker health and job well-being.^[2]^ The construction industry, with its challenging working conditions, unhealthy work environments, and prolonged separation from family life, has garnered significant attention from all sectors of society. As a result, construction workers’ pursuit of health and happiness is often more intense than that of workers in other industries. Job Well-being is a positive emotional response or experience that profoundly affects the physical and mental health of construction workers and has become one of the key indicators for evaluating the construction industry.^[3]^ Therefore, exploring the job well-being of workers within the framework of construction safety is of vital importance for promoting the healthy and sustainable development of the industry.

Currently, there is a wealth of research on the factors influencing employees’ job well-being. Carvajal-Arango et al^[4]^ explored the factors and dimensions of job well-being from the perspective of construction workers, finding that the physical work environment (temperature, lighting, noise, etc) significantly affects their job well-being. Chia-Hao and Ting-Ya^[5]^ discovered that good interpersonal relationships help construction workers establish positive connections with others, foster teamwork, enhance work efficiency, and reduce conflict and stress in the workplace, thereby improving individual well-being. Additionally, factors such as job well-being,^[6]^ job security,^[7]^ and occupational motivation^[8]^ also significantly influence the job Well-being of construction workers. Some scholars have found that when the factors affecting job Well-being are not met, individuals are more likely to fall into unfavorable physiological and psychological states, leading to unsafe behaviors. Valente and Berry et al^[9]^ suggested that increased working hours can lead to heightened negative emotions and psychological stress, which in turn can give rise to anxiety and burnout issues. Moreover, excessively long working hours may also increase fatigue levels among employees, leading to a higher frequency of unhealthy behaviors (such as smoking and alcohol abuse), which can, in severe cases, trigger various health issues. Ganster and Victor^[10]^ indicated that a lack of basic social support and activities may result in psychological problems such as mental stress, emotional depression, and diminished self-confidence, adversely affecting physical and mental health. There exists a direct correlation between the job well-being of construction workers and their unsafe conditions; thus, an in-depth exploration of the relationship between the 2 can help reduce the incidence of safety accidents at its root.

To this end, this paper first analyzes the intrinsic relationship between construction workers’ job well-being and their unsafe state (both psychological and physiological states) from the perspective of Maslow hierarchy of needs. It establishes a framework for understanding the impact of job well-being on unsafe state and reveals the significance of job well-being within the field of construction safety. Furthermore, an indicator system for the factors influencing construction workers’ job well-being is constructed based on dimensions such as living conditions, working conditions, rights protection, safety development (stress), and career advancement. The DEMATEL-ISM model is employed to identify key influencing factors and to uncover the hierarchical structural relationships among these factors. The research results aim to provide recommendations and strategies for enhancing construction workers’ job well-being and reducing unsafe behaviors.

## 2. Relevant concepts and theoretical foundation

### 2.1. Job well-being

(1) Concept

Currently, the concept of happiness has evolved from a general viewpoint into a metric for global well-being and has been integrated into human development analytical frameworks. It has become a significant agenda for governments, international organizations, healthcare enterprises, and research institutions, with the extension of happiness into the work environment emerging as a hot topic of research.^[11]^ Understanding happiness within the context of work offers notable advantages, primarily because there is often a strong correlation between happiness and the inherent prerequisites of work. This understanding helps to delve deeper into how the characteristics of work affect employees’ sense of well-being.^[12]^ In reality, employees with high levels of happiness can bring numerous benefits to organizations; from a more humanistic perspective, individual happiness is crucial for a healthy life. In this context, the construction industry, characterized by intensive labor use, holds significant potential for enhancing employee happiness. The working environment of construction workers exhibits unique characteristics, such as limited technological advancement, a high proportion of less-educated workers, low wages, long working hours, harsh conditions, and severe social deprivation. Performing psychologically and physiologically demanding tasks in dangerous environments greatly impacts employees’ productivity, safety, personal well-being, and work quality. Additionally, work-related diseases rank highly among occupational groups in the construction industry, presenting challenges for effectively promoting occupational health within this sector.

(2) Influencing factors

According to Maslow hierarchy of needs theory, human needs are categorized into 5 levels, ranging from low to high: physiological, safety, social, esteem, and self-actualization. Based on evolutionary theory and organism theory, it can be argued that the existence of these 5 levels of needs is universal in reality.^[13]^ When an individual’s needs are met, they often experience a positive emotional response, referred to as happiness. The inherent logical consistency between Maslow Hierarchy of Needs theory and the job well-being of construction workers is applicable in the study of workers’ happiness. For construction workers, job well-being can be understood as the total utility they perceive from their work and the various impacts arising from it, which includes physiological utility, safety utility, social utility, esteem utility, and self-actualization utility, corresponding to Maslow 5 levels of needs. Given that different construction workers have varying subjective evaluations of the total utility derived from each dimension, a power utility function is chosen as the foundational utility function to establish the total utility function for construction workers’ happiness.


$$\begin{array}{l} M=f(M^{w},M^{A},M^{S},M^{G},M^{N})=X{\left(M^{w}\right)}^{\alpha }{\left(M^{A}\right)}^{\beta }{\left(M^{S}\right)}^{\gamma }{\left(M^{G}\right)}^{\delta }{\left(M^{N}\right)}^{\varepsilon } \end{array}$$ (1)

In this formula, *M* represents the total utility perceived by construction workers from their work and the various impacts arising from it. The terms $M^{w}$, $M^{A}$, $M^{S}$, $M^{G}$, and $M^{N}$ respectively denote the physiological utility, safety utility, social utility, esteem utility, and self-actualization utility. The parameters α, β, γ, δ, and ε represent the subjective evaluations of construction workers regarding the utility of each dimension, with values ranging from 0 to 1.

Physiological needs represent the lowest and most fundamental level in Maslow hierarchy of needs, primarily encompassing material needs closely related to daily life, such as clothing, food, shelter, and transportation. Construction workers typically fall within the age range of 30 to 50 years, often operating within a relatively narrow scope of work and life. The physiological needs of construction workers can be reflected in their living conditions, which encompass both personal and family dimensions. In terms of personal life, the focus is on aspects such as workers’ nutrition, physical and mental health, sleep, and recreational activities. Family life, on the other hand, revolves around workers’ marital status, child-rearing situations, family harmony, and the frequency of family reunions. Grevenstein et al^[14]^ explored the bidirectional relationship between the quality of family relationships, health-related individual differences, psychological distress, and overall happiness. Their research found that the family environment significantly influences psychological health, quality of life, and personal adaptation and happiness. Weiß et al^[15]^ used dimensions such as family happiness, psychological health, sleep quality, and physical health to characterize the subjective well-being of construction workers. The study indicated that negative issues in family atmosphere, sleep conditions, and health status could impose substantial psychological pressure on construction workers, resulting in adverse psychological states and decreased happiness. The spillover theory posits that the values, emotions, behaviors, and skills developed in the workplace can transfer to the home environment and vice versa.^[16]^ Work-family conflict is a form of negative spillover, where tensions and negative emotions from the work (or family) domain affect performance in the family (or work) domain. Compensation theory complements the spillover theory by suggesting that individuals often seek well-being in one area to compensate for unhappiness in another.^[17]^ Generally, individuals may reduce their investment in unsatisfactory domains to increase their investment in potentially satisfying areas. This implies that individuals will reallocate their time and attention between unsatisfactory and potentially satisfying domains, indicating that the tension between family and work clearly affects their sense of happiness.

In contrast to physiological needs, safety needs occupy a higher level in Maslow hierarchy of needs. For construction workers, the connection between safety needs and job well-being is particularly strong due to their prolonged exposure to high-risk environments. This study focuses on issues related to personal safety, safety assurance, and safety development in the work context of construction workers, explaining their safety needs through 3 dimensions: working conditions, rights protection, and safety development. In the dimension of working conditions, this study primarily addresses issues related to construction workers’ working hours, work pressure, working environment, job stability, and income. These factors directly impact construction workers’ work experience and quality of life, making them crucial for understanding workers’ safety needs. Regarding the rights protection dimension, the focus is on social and safety security issues for construction workers, including whether workers have access to legal labor protections, social insurance, compensation for work-related injuries, and the distribution of protective equipment. In the dimension of safety development, the study examines 2 levels: the safety behavior of construction workers and the level of safety management within projects. This includes construction workers’ safety awareness, skills, and habits in their work, as well as the emphasis placed on safety management during project implementation. Existing research indicates a certain correlation between work pressure and job well-being, suggesting that sustained high levels of work pressure can negatively affect an individual’s subjective well-being.^[18]^ Green and Leeves^[19]^ investigated the relationship between family, income, labor dynamics, and happiness in Australia, finding that low wages, job instability, and economic uncertainty all adversely impact happiness. Sleet and Mercy^[7]^ argued that social and safety protections can provide a fundamental sense of security and stability in life, thus contributing to improved well-being. Mearns et al^[20]^ posited that by adopting appropriate safety behaviors and implementing effective project safety management measures, workers can reduce the likelihood of accidents, enhance work efficiency and teamwork, and gain more opportunities for career development and skill enhancement, thereby increasing their overall happiness.

Social needs, esteem needs, and self-actualization needs are regarded as higher-level human needs. This study primarily considers the career advancement conditions of construction workers, focusing on 3 dimensions: interpersonal relationships, self-esteem, and occupational motivation. Interpersonal relationships refer to the interactions between construction workers and their colleagues, managers, and family members. Positive interpersonal dynamics are crucial, as they can significantly enhance workers’ sense of belonging within the workplace and provide essential support and assistance, ultimately improving job well-being and overall happiness. Haar et al,^[21]^ in their research on employee well-being in New Zealand, found that interpersonal relationships in the workplace hold significant importance for employee happiness. Self-esteem is the recognition and affirmation of construction workers’ professional identity and value. It encompasses their pride in their skills and knowledge, as well as acknowledgments of their contributions to the construction industry. When construction workers positively assess their professional identity and feel respected and valued in their work, they are more likely to experience positive emotional states.^[22]^ Occupational motivation serves as a key driving force for construction workers in pursuing career advancement. When workers possess strong occupational motivation, they exhibit positive attitudes and behaviors, actively seeking better career opportunities. By continually learning and enhancing their skill levels, workers can achieve improved career prospects and compensation, thereby increasing their overall happiness.^[8]^

### 2.2. Unsafe state

(1) Concept

Unsafe state describe a situation in which individuals and objects are exposed to various risk factors that could trigger personal injury events or lead to property loss.^[23]^ Based on Heinrich accident causation theory, both unsafe human behaviors and unsafe conditions of objects are direct triggers for accidents. Research indicates that the majority of industrial injury incidents are caused by human factors.^[24]^ In the “Swiss Cheese” model, James Reason categorizes the human factors leading to accidents into explicit and implicit causes. Explicit causes refer to unsafe behaviors that have a direct connection to the occurrence of accidents, while implicit causes (also known as latent failures) are potential hazards hidden within the organizational system. These implicit factors do not directly lead to accidents until they are uncovered following an incident.^[25]^ The unsafe condition of individuals parallels the role of implicit causes in the accident occurrence process within the “Swiss Cheese” model. It manifests as latent dangers within the dimensions of time and space, which are often difficult to detect and intervene in. Typically, they occupy a subjective central role in the accident chain, reflecting the complexity and uncertainty of the interactions between human behavior, decision-making, and the environment.^[26]^

(2) Influencing factors

Building on Reason’s “Swiss Cheese” model, Scott A. Shappell and Douglas A. Wiegmann introduced the Human Factors Analysis and Classification System to delve deeper into analyzing human behavior and decision-making within complex systems, aiming to identify and prevent potential safety issues arising from human factors.^[27]^ Within the human factors analysis and classification system framework, the prerequisites for unsafe behaviors include direct causes that lead to their occurrence, comprising personnel factors, operator states, and environmental factors. Notably, substandard operator states can also be referred to as human unsafe behavior states. For construction workers, unsafe conditions typically fall into 2 main categories: First, adverse physiological states: These refer to pathological or physiological conditions that hinder safe operations, such as mental fatigue, muscle fatigue, and poor physiological perception (e.g., visual or auditory impairments). Second, adverse psychological states: These pertain to mental states that can affect the execution of work tasks, including mental fatigue, loss of situational awareness, low mood, high psychological pressure, and diminished self-confidence. Both categories of unsafe states can impair workers’ judgment, reaction speed, and work efficiency, thereby increasing the risk of accidents. Chen et al^[28]^ identified that states such as mental fatigue, illness, and physiological fatigue can trigger unsafe behaviors among miners. Parviniannasab et al^[29]^ validated a structural equation model on miner’ negative emotional states, concluding that students’ unsafe behaviors were indirectly influenced by factors like personality and attention. Jue and Ha^[30]^ through eye-tracking and behavioral response experiments, demonstrated that adverse physiological and psychological states significantly negatively impacted miners’ reaction capabilities and could directly lead to unsafe behaviors. The unsafe behaviors of workers are contingent upon their physiological and psychological states; poor physiological and psychological conditions can directly influence unsafe behaviors, jeopardizing the safety of production systems and work groups.

(3) Causes

Maslow hierarchy of needs theory holds significant applicability in addressing the issue of unsafe conditions among construction workers. The physiological, safety, social, esteem, and self-actualization needs of construction workers are all closely linked to their job performance, safety awareness, and accident risk. To enhance construction workers’ safety awareness and reduce the occurrence of unsafe accidents, it is essential to recognize and satisfy their varying levels of needs.

Physiological needs: when construction workers’ physiological needs are unmet, such as poor nutrition, deteriorating health, insufficient rest, and strained family relationships, these factors negatively impact their perceptual abilities, physical strength, and mental acuity. Consequently, this leads to increased psychological stress, distracted attention, and emotional instability, thereby raising the risk of safety accidents. Safety needs: unmet safety needs can impair workers’ attention, increase psychological stress, and destabilize emotions. An unsafe working environment and lack of safety measures can divert their attention, resulting in decreased work efficiency and increased accident risks. Furthermore, concerns and anxieties regarding safety may create psychological pressure, adversely affecting job performance and health. Additionally, unmet safety needs can lead to emotional instability, irritability, or depression, further impacting work performance and interpersonal relationships. Social needs: a lack of basic social support and engagement in social activities among construction workers can trigger a range of psychological issues, including heightened psychological stress, emotional depression, and diminished self-confidence. These adverse factors significantly affect their mental health and overall well-being. Esteem needs: unmet esteem needs can lead to feelings of sadness, anger, and anxiety among construction workers, increasing psychological stress and diminishing their self-confidence at work. This, in turn, affects their quality of life and decision-making abilities. Self-actualization needs: self-actualization needs pertain to construction workers’ desires for personal career development and the realization of personal goals. If these needs are unmet, workers may experience negative emotions that hinder their enthusiasm and efficiency at work. Additionally, the inability to achieve career advancement or personal goals can lead workers to feel inadequate in terms of their abilities or value, which can diminish their self-confidence and negatively impact their job performance and risk perception.

**Figure 1.**
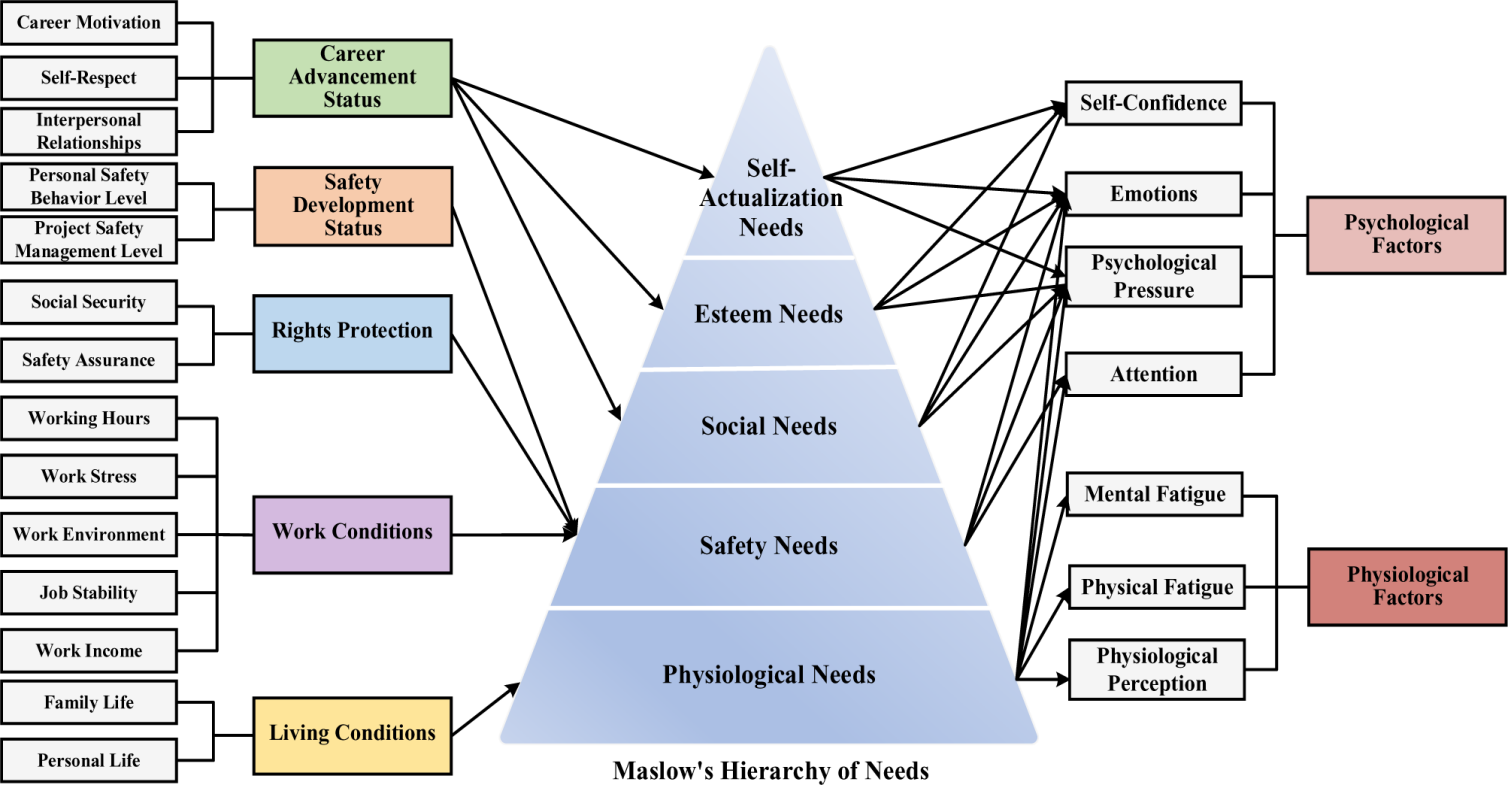
Influence framework of construction workers’ job well-being and unsafe state: using Maslow Hierarchy of Needs as a “Bridge”.

In summary, this study utilizes Maslow hierarchy of needs theory as a “bridge” to construct a framework illustrating the influence of job well-being and unsafe conditions (both physiological and psychological factors) among construction workers, as depicted in Figure 1.

## 3. Research method

### 3.1. DEMATEL-ISM method

**Figure 2.**
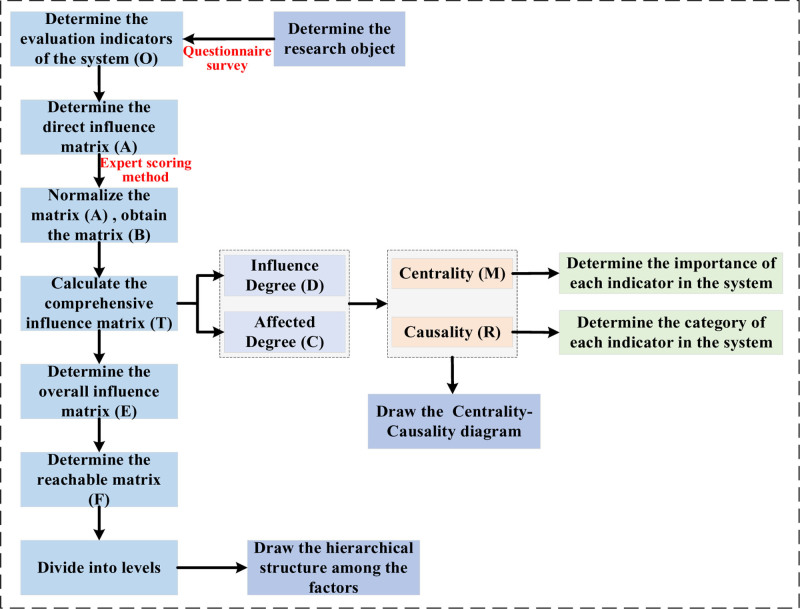
Implementation flowchart of the DEMATEL-ISM method. DEMATEL-ISM = decision-making trial and evaluation laboratory – interpretative structural modeling method.

The decision-making trial and evaluation laboratory (DEMATEL) model can use matrix operations to calculate the causal relationships and influence intensities among factors.^[31]^ By visualizing the causal relationships among factors, it can reveal the key influencing factors and the degree of influence in complex problems. However, this method cannot effectively identify the hierarchical structure of factors in the system.^[32]^ The interpretative structural modeling method (ISM) method analyzes the direct binary correlation relationships among the subsystems (factors or elements) that constitute the system.^[33]^ Based on operations such as Boolean algebra, it constructs a multi-level hierarchical directed topological graph, but it cannot determine the degree of influence of elements on the system.^[34]^ Therefore, by combining the 2 methods, it is possible to identify the key elements in the system and their degree of influence, and also to construct the hierarchical structure of the system elements. It is worth noting that as an empirical analysis, this study adopts the DEMATEL-ISM method to explore causal relationships and hierarchical structures among factors. Unlike systematic reviews or meta-analyses, our approach focuses on primary data collection and model-based analysis. The implementation flowchart of the DEMATEL-ISM method is shown in Figure 2.

### 3.2. Influencing indicators

Based on the aforementioned theoretical framework, exploring the factors that influence construction workers’ job well-being is crucial. To ensure the scientific rationality of the selected indicators, a literature review was conducted, followed by consultation interviews with 10 researchers. The interview subjects included construction workers from various construction sites in Hubei Province. In accordance with the theoretical foundation and the results from the interviews, a survey questionnaire was designed and refined. The questionnaire was distributed in an offline format, with a total of 200 copies issued and 188 valid responses collected, resulting in an effective response rate of 94%. Among the respondents, 96.2% were male and 3.8% were female; 22.3% were aged 20 to 30, 38.3% were aged 31 to 40, 34.6% were aged 41 to 50, and 4.8% were over 50; in terms of marital status, 19.7% were unmarried, 78.5% were married, and 1.8% were divorced. Regarding educational background, 72.3% had completed junior high school or lower, 25.5% had completed high school or vocational training, and 2.2% had obtained an associate degree or higher. For work experience, 10.6% had <3 years, 73.9% had 3 to 10 years, and 15.5% had over 10 years. The age, educational background, and work experience of the surveyed individuals aligned with the basic characteristics of construction workers, ensuring the universality and authenticity of the survey data.

To ensure the effectiveness of the selected indicators, SPSS software (Chicago) was employed to conduct reliability and validity analyses on the 188 valid questionnaires collected. The internal consistency was tested using Cronbach α coefficient, with results indicating that the reliability coefficients for the overall questionnaire and each dimension were >0.7, demonstrating good reliability and stability. An exploratory factor analysis was performed to assess the structural validity of the questionnaire. The results showed a KMO measure value >0.8 and a significance level of *P* < .001, both of which fall within effective ranges, indicating a high correlation among the variables and validating the overall questionnaire. As a result, this study ultimately integrated and confirmed the construction of an indicator system comprising 5 primary indicators and 14 secondary indicators that affect construction workers’ job well-being. The index system and its sources are shown in Table 1.^[7,9,14,18–22,35–37]^

**Table 1:** Indicator system for factors affecting construction workers’ job well-being.

Primary indicators	Secondary indicators	Explanation	Source
Living conditions	P1 personal life	A comprehensive evaluation of an individual’s daily life behaviors, including work, leisure, social interactions, entertainment, and physical functions.	Lingard^[35]^
	P2 family life	The living conditions and circumstances of family members, encompassing aspects such as family member composition, living environment, economic status, and social environment.	Grevenstein^[14]^
Employment conditions	P3 work income	The compensation obtained from engaging in actual work, including wages, bonuses, allowances, and other forms of remuneration.	Green^[19]^
	P4 job stability	The ability of workers to maintain stable employment over a period while receiving compensation that allows them to sustain a relatively stable living standard based on their labor contributions.	Yun^[36]^
	P5 work environment	The physical and social environments in which an individual operates during work.	Lingard^[35]^
	P6 work stress	A negative response experienced by individuals in the work environment due to various physical, psychological, and social pressures.	Amponsah^[18]^
	P7 working hours	The legally stipulated duration that workers are required to engage in labor within a day and a week.	Valente and Berry^[9]^
Rights protection	P8 safety assurance	A series of measures implemented to ensure that workers are protected from the risks of accidental injuries during work, safeguarding their life and health.	Sleet^[7]^
	P9 social security	The insurance system established by the state through legislation that actively mobilizes resources from various sectors of society to provide coverage for workers against risks of accidents, illnesses, unemployment, and maternity during production.	Sleet^[7]^
Safety and development (stress) conditions	P10 personal safety behavior level	The behaviors of workers during production, including adherence to safety regulations and operational procedures, correct use of personal protective equipment, and avoidance of hazardous operations to ensure their own safety and that of others.	Mearns^[20]^
	P11 project safety management level	The capacity of an enterprise to identify, assess, control, and monitor potential safety risks during project implementation. This includes formulating safety management plans, establishing safety management systems, ensuring safety equipment, improving safety protocols, conducting safety education and training, and addressing safety issues in a timely manner.	Mearns^[20]^
Career advancement conditions	P12 interpersonal relationships	The direct psychological connections formed between individuals through interaction and mutual influence.	Haar^[21]^
	P13 self-respect	An individual’s self-perception filled with confidence and esteem, manifested as recognition and respect for one’s own value, abilities, and dignity.	Teixeira^[22]^
	P14 career motivation	The psychological processes that drive and guide workers in their occupational activities, enabling them to maintain their professional endeavors to achieve specific career objectives.	Dipboye^[37]^

## 4. Results

### 4.1. Construction of the DEMATEL-ISM model

Based on the indicator system of factors influencing work happiness presented in Table 1, a survey questionnaire was designed for experts. Twelve experts in the fields of safety behavior and social psychology were invited to participate in the survey, consisting of 6 professors and doctoral students, as well as 6 managers from construction enterprises. The scoring employed a 5-point Likert scale, with ratings corresponding to: no impact (0 points), minor impact (1 point), moderate impact (2 points), significant impact (3 points), and extreme impact (4 points). The initial scores given by the experts were averaged arithmetically to obtain the direct influence matrix A=[$x_{ij}$]_n×n_, where x_*ij*_ indicates the degree of influence of factor *i* on factor *j*, as shown in Table 2.

**Table 2:** Direct influence matrix.

Factors	A1	A2	A3	A4	A5	A6	A7	A8	A9	A10	A11	A12	A13	A14
A1	0	3.17	2.5	3.17	0	2	1.33	1	1	3.33	0.5	3.17	2.83	3
A2	3.83	0	2	3.17	0	2.83	1.33	0.83	0.83	3.17	0.5	3	2.83	3
A3	3.83	3.83	0	3.67	1	2.83	3.67	0	0.83	3	0.83	3.33	3	3.33
A4	3.5	3.33	1.83	0	1	2	1.17	0	0	3.17	0	3	2.83	3
A5	3.5	3.83	1.67	3.17	0	3.83	3.67	1.17	0.67	2.83	0.83	1	0.83	2.67
A6	3.67	3.67	1.83	3.67	0	0	2.17	1	1	3	1	2.83	1.83	2.67
A7	3.67	3.67	2	3.83	0	3.5	0	0.67	0	2.83	1.33	3.33	1.83	2.83
A8	3.83	3.83	1	2.83	0	3.83	3.67	0	0.83	3.67	3.33	1	1.33	0.83
A9	3.83	3.5	1	3.17	0	3.17	0.67	1	0	2	2	0	1.17	0.83
A10	2	2	0.67	1	0.67	1.33	0.67	0	0	0	1.67	2.83	1	1.83
A11	1	1	0	3.5	0	3.33	0	0.67	0.67	2.83	0	0	0	0
A12	3.33	2.83	0.67	0	0	3.33	2.83	0	0	3.83	0	0	2.83	2.83
A13	2	1.67	0.5	0.83	0	1.17	0.67	0	0	2	0	2.5	0	2.5
A14	2	1.83	1	1.17	0.5	1	1	0.5	0.5	1.83	0.83	2	2.33	0

Normalization of matrix A yields matrix B, ensuring that each element in matrix B is confined within the range [0,1]. Subsequently, the comprehensive influence matrix T=[${t_{i}}_{j}$] is calculated. Using Matlab, the standardized influence matrix is computed and converted into the comprehensive influence matrix T, as shown in Table 2. Based on formulas (4–6), the influence degree D, affected degree C, centrality M, and causality R values for each factor are calculated, allowing for the assessment of the mutual influence among factors, as presented in Table 3.

**Table 3:** Comprehensive influence matrix.

Factors	P1	P2	P3	P4	P5	P6	P7	P8	P9	P10	P11	P12	P13	P14
P1	0.238	0.309	0.261	0.308	0.045	0.276	0.243	0.095	0.094	0.326	0.127	0.382	0.355	0.354
P2	0.324	0.231	0.244	0.307	0.044	0.295	0.241	0.089	0.087	0.321	0.124	0.376	0.352	0.353
P3	0.349	0.347	0.195	0.343	0.076	0.319	0.315	0.069	0.088	0.341	0.139	0.399	0.365	0.379
P4	0.299	0.293	0.232	0.197	0.078	0.256	0.227	0.056	0.054	0.302	0.097	0.357	0.343	0.337
P5	0.340	0.347	0.247	0.340	0.046	0.345	0.325	0.107	0.088	0.333	0.146	0.333	0.300	0.362
P6	0.323	0.321	0.237	0.326	0.044	0.220	0.266	0.095	0.092	0.319	0.140	0.370	0.318	0.323
P7	0.329	0.327	0.246	0.335	0.045	0.321	0.201	0.086	0.063	0.322	0.150	0.392	0.325	0.357
P8	0.331	0.330	0.213	0.315	0.042	0.332	0.309	0.068	0.091	0.340	0.310	0.315	0.297	0.286
P9	0.286	0.279	0.190	0.284	0.035	0.271	0.184	0.100	0.055	0.254	0.177	0.229	0.252	0.237
P10	0.202	0.200	0.151	0.175	0.068	0.186	0.158	0.046	0.044	0.163	0.170	0.270	0.204	0.226
P11	0.136	0.136	0.082	0.308	0.020	0.300	0.089	0.092	0.090	0.184	0.059	0.124	0.113	0.113
P12	0.271	0.259	0.176	0.184	0.034	0.270	0.272	0.052	0.049	0.293	0.092	0.236	0.315	0.306
P13	0.168	0.160	0.120	0.133	0.023	0.145	0.130	0.031	0.030	0.175	0.056	0.227	0.135	0.215
P14	0.214	0.209	0.178	0.190	0.058	0.187	0.180	0.074	0.072	0.218	0.124	0.258	0.264	0.181

$$\begin{array}{l} B=\frac{A}{A_{\max}}=\frac{A}{\max(\max\sum_{i=1}^{n}x_{ij},\max\sum_{j=1}^{n}x_{ij})} \end{array}$$ (2)

$$\begin{array}{l} T=(B+B^{2}+B^{3}+\ldots +B^{k})=\sum_{k=1}^{\infty }B^{k}=B{\left(I-B\right)}^{-1} \end{array}$$  (3)

$$\begin{array}{l} D_{i}=\sum_{j=1}^{n}{t_{i}}_{j}(i=1,2,3\ldots ,n) \end{array}$$ (4)

$$\begin{array}{l} C_{i}=\sum_{i=1}^{n}{t_{i}}_{j}(\;j=1,2,3\ldots ,n) \end{array}$$ (5)

$$\begin{array}{l} M_{i}=D_{i}+C_{i};R_{i}=D_{i}-C_{i} \end{array}$$ (6)

Based on the comprehensive influence matrix T, the overall influence matrix E(E=[$e_{ij}$]_n×n_]) is obtained by adding the identity matrix I. Using formula (7), we can calculate λ = 0.30, where the mean $\bar{x}$ and standard deviation σ are derived from all elements in the comprehensive matrix T, allowing us to exclude the interference from weaker influencing factors. Combining this with formula (8), the elements in matrix E are processed to yield the reachable matrix F(F = [$f_{ij}$]_n×n_]), as shown in Table 4.

**Table 4:** Reachable matrix.

Factors	P1	P2	P3	P4	P5	P6	P7	P8	P9	P10	P11	P12	P13	P14
P1	1	1	0	1	0	0	0	0	0	1	0	1	1	1
P2	1	1	0	1	0	1	0	0	0	1	0	1	1	1
P3	1	1	1	1	0	1	1	0	0	1	0	1	1	1
P4	1	1	0	1	0	0	0	0	0	1	0	1	1	1
P5	1	1	0	1	1	1	1	0	0	1	0	1	0	1
P6	1	1	0	1	0	1	0	0	0	1	0	1	1	1
P7	1	1	0	1	0	1	1	0	0	1	0	1	1	1
P8	1	1	0	1	0	1	1	1	0	1	1	1	1	0
P9	0	0	0	0	0	0	0	0	1	0	0	0	0	0
P10	0	0	0	0	0	0	0	0	0	1	0	0	0	0
P11	0	0	0	1	0	1	0	0	0	0	1	0	0	0
P12	0	0	0	0	0	0	0	0	0	1	0	1	1	1
P13	0	0	0	0	0	0	0	0	0	0	0	0	1	0
P14	0	0	0	0	0	0	0	0	0	0	0	0	0	1

$$\begin{array}{l} \lambda =\bar{x}+\sigma \end{array}$$ (7)

$$\begin{array}{l} {f_{i}}_{j}=\left\{ \begin{array}{ll} & 1,e_{ij}\geq \lambda (i,j=1,2\ldots n)\;\\ & 0,e_{ij}\leq \lambda (i,j=1,2\ldots n)\; \end{array}\right. \end{array}$$ (8)

Based on the reachable matrix *F*, the reachable set *R* and the antecedent set *Q* of factors influencing the happiness of construction workers are derived using formulas (9) and (10). Subsequently, the hierarchical classification of influencing factors is conducted based on the intersection conditions as per formula (11).

$$\begin{array}{l} R(x_{i})=\left\{x_{i}|F_{ij}=1\right\}(i=1,2,3\ldots n) \end{array}$$ (9)

$$\begin{array}{l} Q(x_{i})=\left\{x_{i}|F_{ij}=1\right\}(\;j=1,2,3\ldots n) \end{array}$$ (10)

$$\begin{array}{l} R(x_{i})\cap Q(x_{i})=R(x_{i}) \end{array}$$ (11)

**Figure 3.**
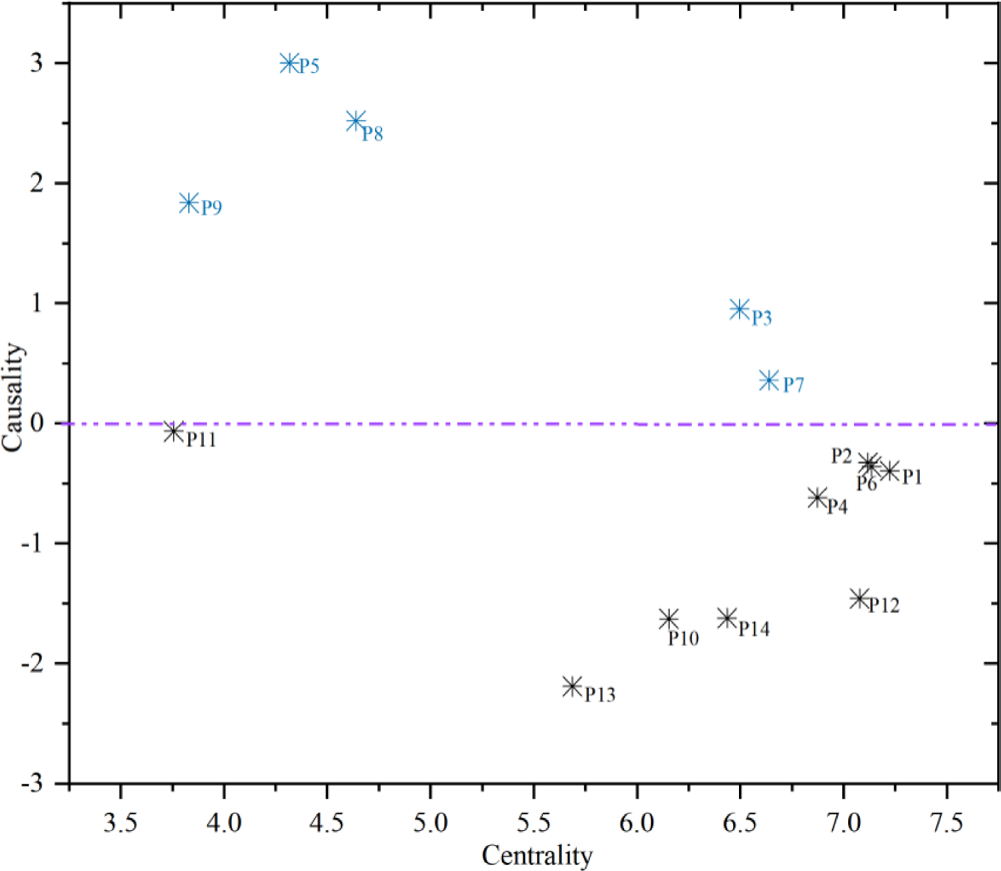
Two-dimensional coordinate diagram of centrality-causality.

To visually present the importance of various influencing factors and the relationships between their influences and being influenced, a cause-and-effect relationship diagram of the influencing factors is illustrated in Figure 3.

### 4.2. Centrality analysis

The centrality Mi is defined as the sum of influence degree Di and affected degree Ci, essentially indicating the criticality of influencing factor i within the network. A larger centrality i for an influencing factor Mi signifies a greater direct impact on the happiness of construction workers. Based on Table 5 and Figure 2, the top 5 factors ranked by centrality Mi are P1 (personal life), P2 (family life), P6 (work stress), P12 (interpersonal relationships) and P4 (job stability), with centrality values of 7.223, 7.136, 7.117, 7.077, and 6.873, respectively. From the perspective of their respective dimensions, among the top 10 factors ranked by centrality, 2 belong to the dimension of living conditions, 2 to the dimension of employment conditions, and one to the dimension of career advancement. This indicates that influencing factors related to the living conditions, work conditions, and career advancement of construction workers have a strong direct impact on their work happiness.

**Table 5:** DEMATEL calculation results.

Influencing factors	Influence degree		Affected degree		Centrality		Causality		Attributes
	Values	Ranking	Values	Ranking	Values	Ranking	Values	Ranking	
P1	3.413	5	3.810	5	7.223	1	−0.397	11	▲
P2	3.388	7	3.748	6	7.136	2	−0.36	12	▲
P3	3.724	1	2.772	10	6.496	7	0.952	4	●
P4	3.128	8	3.745	7	6.873	5	−0.617	10	▲
P5	3.659	2	0.658	14	4.317	12	3.001	1	●
P6	3.394	6	3.723	8	7.117	3	−0.329	13	▲
P7	3.499	4	3.140	9	6.639	6	0.359	5	●
P8	3.579	3	1.060	12	4.639	11	2.519	2	●
P9	2.833	9	0.997	13	3.83	13	1.836	3	●
P10	2.263	12	3.891	4	6.154	9	−1.628	7	▲
P11	1.846	13	1.911	11	3.757	14	−0.065	14	▲
P12	2.809	10	4.268	1	7.077	4	−1.459	9	▲
P13	1.748	14	3.938	3	5.686	10	−2.190	6	▲
P14	2.407	11	4.029	2	6.436	8	−1.622	8	▲

● denotes causal factors, and ▲ denotes outcome factors.

DEMATEL = Decision-making Trial and Evaluation Laboratory.

### 4.3. Causality analysis

The causality Ri is defined as the difference between the influence degree Di and the affected degree Ci, essentially indicating the contribution of influencing factor i to the overall network of influencing factors. If the causality Ri >0, the influencing factor i is classified as a causal factor. A larger causality i for a causal factor Ri suggests that this factor is more likely to influence other factors. According to Table 5 and Figure 2, the top 5 causal factors are P5 (Work Environment), P8 (Safety Assurance), P9 (Social Security), P3 (Work Income) and P7 (Working Hours), with causalities of 3.001, 2.519, 1.836, 0.952, and 0.359, respectively. From the perspective of their respective dimensions, 2 of these influencing factors belong to the dimension of rights protection, while 3 belong to the dimension of work conditions. This indicates that the dimensions of rights protection and work conditions have a strong indirect impact on the happiness of workers. If the causality Ri <0, the influencing factor i is classified as an outcome factor. A smaller causality i for an outcome factor Ri indicates that this influencing factor is more susceptible to the influence of other factors. The top 5 outcome factors are P13 (Self-Respect), P10 (Personal Safety Behavior Level), P14(Career Motivation), P12 (Interpersonal Relationships) and P4 (Job Stability), with causalities of −2.190, −1.628, −1.622, −1.459, and −0.617, respectively. These influencing factors exhibit considerable uncertainty and are easily affected by various other factors. Therefore, in enhancing the work happiness of construction workers, these should be regarded as uncertainty factors and considered comprehensively. A diversified strategy should be adopted, including multiple approaches such as increasing work income, improving work environment, alleviating work stress, enhancing workers’ safety behavior levels, and boosting career motivation.

### 4.4. Key influencing factors

In identifying the key influencing factors, the focus is primarily on the centrality and causality of each influencing factor, while also considering their influence degree and affected degree. Based on the identification criteria for key influencing factors established by Yong,^[38]^ the specific criteria for this study are as follows: key influencing factors should be ranked among the top 5 in terms of centrality or classified as causal factors in the top 5 or as outcome factors in the top 5. Key influencing factors should also be ranked among the top 5 in terms of influence degree or affected degree. Key influencing factors must simultaneously satisfy both criteria. According to Table 5 and the aforementioned identification criteria for key influencing factors, this study identifies a total of 9 key influencing factors affecting work happiness, which are personal life (P1), work income (P3), work environment (P5), work stress (P6), working hours (P7), safety assurance (P8), personal safety behavior level (P10), interpersonal relationships (P12), self-respect (P13) and career motivation (P14). From the perspective of the primary indicators associated with these 9 key factors, there is 1 factor from the dimension of living conditions, 1 from the dimension of rights protection, and 1 from the dimension of safety development. Additionally, there are 3 factors from the dimension of career advancement and 4 from the dimension of work conditions. This indicates that work conditions and career advancement have a significant impact on the happiness of construction workers.

**Figure 4.**
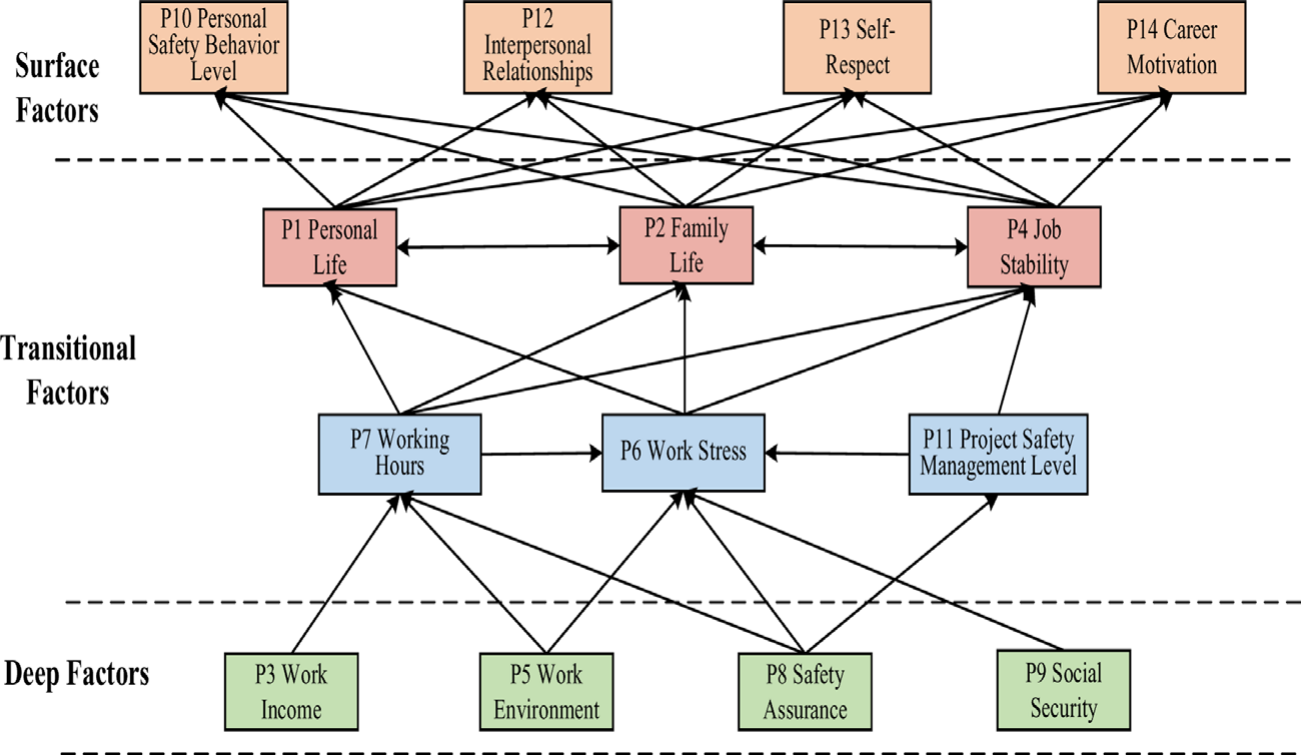
ISM model of influencing factors of construction workers’ happiness at work. ISM = interpretative structural modeling method.

From the results of the ISM analysis (Fig. 4), it is evident that the influencing factors affecting the work happiness of construction workers exhibit complex relationships, including same-level and cross-level connections. In the ISM model, the influencing factors on the happiness of construction workers are divided into 3 major factor sets: surface factors (level 1): P10 (personal safety behavior level), P12 (interpersonal relationships), P13 (self-respect) and P14 (career motivation); transitional factors: P1 (personal life), P2 (family life), P4 (job stability), P6 (work stress), P7 (work environment) and P11 (project safety management level). These factors belong to levels 2 and 3 in the ISM model. Transitional factors are influenced by deeper factors and simultaneously induce the emergence of surface factors. Deep factors (level 4): P3 (work income), P5 (working hours), P8 (safety assurance) and P9 (social security).

Therefore, multiple factors such as living conditions, work conditions, safety development (stress), and career advancement interact and influence each other, collectively constituting an individual’s overall happiness and safety behavior level within the construction industry. In the ISM model, factors with a higher degree of connectivity include P3 (work income), P8 (safety assurance), P5 (work environment), P7 (working hours), and P6 (work stress), which align with the key influencing factors identified by DEMATEL and warrant sufficient attention.

## 5. Discussion

This study reveals the intrinsic correlation between the job well-being of construction workers and their unsafe conditions, and identifies the key influencing factors and their hierarchical relationships through the DEMATEL-ISM model.

The level of personal safety behavior is influenced by multiple dimensions, including living conditions, work conditions, and rights protection. Living conditions encompass various aspects such as workers’ physical and mental health, leisure and recreation, the degree of family harmony, and the frequency of family reunions. These factors directly or indirectly affect an individual’s safety behavior level and significantly impact the work performance and safety behaviors of construction workers. When issues arise in the living conditions of construction workers, such as impaired physical functions, insufficient rest, and an imbalance between work and family life, these adverse factors can negatively affect the individual’s attention, judgment, and behavioral control in the workplace. Elements of the work environment, such as facility conditions, work pressure, working hours, and job stability, also directly influence the safety behaviors of construction workers. For instance, prolonged high-intensity work pressure or an unstable work environment may lead to fatigue and lack of concentration, thereby increasing the risk of unsafe behaviors. Rights protection includes various measures such as labor protection and social insurance, aimed at ensuring the safety and welfare of construction workers at work. If these rights protection measures are not adequately implemented, it may pose a threat to the workers’ safety behavior level. Specifically, when labor protection is insufficient or social insurance is lacking, workers may face greater risks in the workplace, such as occupational accidents and health issues, which can lead to distraction, lack of concentration, or engagement in unsafe behaviors. Moreover, inadequate rights protection may leave workers feeling unsupported and insecure, resulting in a lack of confidence while working and unmet safety needs, further affecting their safety behavior level.

The interpersonal relationships, self-respect, and career motivation of construction workers are also influenced by a combination of factors, including living conditions and work conditions. Interpersonal communication is a vital part of a construction worker’s life, and the relationships among workers are affected by various factors such as social networks, cultural backgrounds, and work relationships. The interactions with colleagues, superiors, and subordinates in the workplace have significant implications for their mental health and work efficiency. When the social status and interpersonal relationships of construction workers are impacted, such as experiencing strained relationships with colleagues, it may lead to a loss of positive interaction and effective communication, ultimately affecting their work performance. Positive interpersonal relationships can enhance job well-being and happiness among workers, as well as promote teamwork and improve productivity. Self-respect is a complex concept influenced by various factors, including an individual’s personality traits, sense of self-efficacy, and life experiences. When construction workers encounter setbacks or difficulties at work and feel disrespected or undervalued, it can trigger doubts about their self-worth and abilities, leading to diminished self-confidence and emotional distress. Career motivation primarily reflects an individual’s aspirations and goals in their work. When construction workers are dissatisfied with their work environment or feel overwhelmed by working hours and job stress, they may exhibit decreased enthusiasm for their jobs, reduced work efficiency, and obstacles to career development.

The findings of this study reveal a direct correlation between construction workers’ job well-being and their unsafe states, with key factors such as income, safety assurance, and work environment playing pivotal roles. To strengthen the generalizability of these findings, we conducted a comparative analysis with prior studies across diverse contexts. For instance, Santana et al^[2]^ explored similar well-being factors among construction workers in the Brazilian Amazon, identifying stress at work and management’s emphasis on safety as critical drivers of unsafe behaviors. While their findings align with our results regarding safety assurance and work stress, differences in cultural and regulatory contexts highlight the need for localized interventions. This comparison underscores the universality of core factors like safety and income while emphasizing contextual nuances in implementation strategies. Additionally, we reviewed control cases from industries with similar risk profiles, such as mining^[28]^ and manufacturing.^[39]^ These studies consistently identified income stability and workplace safety as foundational to worker well-being, reinforcing our conclusions. However, construction workers’ itinerant nature and male-dominated culture introduce unique stressors (e.g., family separation) that differentiate their well-being dynamics from other sectors. By contextualizing our results within broader occupational health literature, we demonstrate both the transferability and specificity of our findings.

## 6. Conclusion

Using Maslow hierarchy of needs as a “bridge,” this paper identifies and analyzes the influencing factors of construction workers’ happiness at work, providing a new perspective and thinking path for understanding the unsafe state and behavior of construction workers. The research results indicate that personal safety behavior level, interpersonal relationships, self-respect, and career motivation are the most direct factors affecting work happiness. In contrast, work income, working hours, safety assurance, and social security are fundamental factors influencing the work happiness of construction workers. It is worth noting that, work income, safety assurance, work environment, working hours, and work stress have a significant impact on construction workers’ happiness. Therefore, the following measures should be taken in future work: ensure the reasonableness and fairness of work income: Develop reasonable wage policies and equitable income distribution mechanisms to stimulate workers’ enthusiasm and job well-being. Strengthen safety assurance measures: enhance safety training, provide safety equipment, and improve safety management at construction sites to ensure the safety and health of workers. Create a good work environment: pay attention to workplace ventilation, lighting, temperature, and noise control to improve workers’ work efficiency and comfort. Arrange working hours reasonably: reduce nighttime construction to enhance workers’ work efficiency and quality of life. Address work stress: Implement effective measures for alleviation and control, such as providing psychological counseling services and conducting health activities to prevent psychological issues and health problems arising from excessive work stress.

This study provides empirical evidence and strategic recommendations for improving construction workers’ job well-being and reducing unsafe behaviors. However, the following limitations should be noted. The limitations of this study mainly lie in the geographical coverage of the sample. The survey subjects are concentrated on construction workers in Hubei Province. However, construction workers in different regions across the country vary significantly in terms of cultural backgrounds, working environments, and educational levels. Therefore, the research conclusions may not fully reflect the overall characteristics of construction workers nationwide. Future research can further expand the sample coverage (for example, by including construction sites in different regions and with different economic levels) to enhance the generalizability of the research findings.

## Author contributions

**Conceptualization:** Qi Liu, Zhengqing Zhong, Yaxin Li.

**Formal analysis:** Qi Liu, Zhengqing Zhong, Yaxin Li.

**Funding acquisition:** Qi Liu.

**Methodology:** Qi Liu, Yaxin Li.

**Software:** Zhengqing Zhong.

**Writing – original draft:** Qi Liu, Zhengqing Zhong, Yaxin Li.

**Writing – review & editing:** Qi Liu, Yaxin Li.
